# A Composite Nanosystem as a Potential Tool for the Local Treatment of Glioblastoma: Chitosan-Coated Solid Lipid Nanoparticles Embedded in Electrospun Nanofibers

**DOI:** 10.3390/polym13091371

**Published:** 2021-04-22

**Authors:** Barbara Vigani, Caterina Valentino, Giuseppina Sandri, Roberta Listro, Francesca Fagiani, Simona Collina, Cristina Lanni, Maria Cristina Bonferoni, Carla M. Caramella, Silvia Rossi, Franca Ferrari

**Affiliations:** Department of Drug Sciences, University of Pavia, Viale Taramelli 12, 27100 Pavia, Italy; barbara.vigani@unipv.it (B.V.); caterina.valentino01@universitadipavia.it (C.V.); giuseppina.sandri@unipv.it (G.S.); roberta.listro01@universitadipavia.it (R.L.); francesca.fagiani@unipv.it (F.F.); simona.collina@unipv.it (S.C.); cristina.lanni@unipv.it (C.L.); cbonferoni@unipv.it (M.C.B.); carla.caramella@unipv.it (C.M.C.); franca.ferrari@unipv.it (F.F.)

**Keywords:** glioblastoma multiforme, solid lipid nanoparticles, coacervation, chitosan-coating, O-carboxymethyl chitosan, electrospinning, composite nanosystem

## Abstract

Glioblastoma multiforme (GBM) is one of the most prevalent and aggressive brain tumors for which there is currently no cure. A novel composite nanosystem (CN), consisting of chitosan-coated Solid Lipid Nanoparticles (c-SLN) embedded in O-carboxymethyl chitosan (O-CMCS)-containing nanofibers (NFs), was proposed as a potential tool for the local delivery of lipophilic anti-proliferative drugs. Coacervation was selected as a solvent-free method for the preparation of stearic acid (SA) and behenic acid (BA)-based SLN (SA-SLN and BA-SLN respectively). BA-SLN, containing 0.75% *w*/*w* BA sodium salt and 3% *w*/*w* poly(vinyl alcohol) (PVA), were selected for the prosecution of the work since they are characterized by the lowest size functional to their subsequent coating and incorporation in nanofibers. BA-SLN were coated with chitosan (CS) by means of a two-step coating method based on the physical absorption of positively charged CS chains on the SLN negative surface. Nile Red (NR), chosen as the hydrophobic model dye, was dissolved in a micellar solution of BA sodium salt and then added with a coacervating solution until pH ≅ 2.5 was reached. Immunocytochemistry analyses highlighted that CS-coated BA-SLN (c-BA-SLN) exhibited a higher accumulation in human glioblastoma cells (U-373) after 6 h than CS-free BA-SLN. Finally, the c-BA-SLN dispersion was blended with a solution consisting of freely soluble polymers (O-CMCS, poly(ethylene oxide) and poloxamer) and then electrospun to obtain NFs with a mean diameter equal to 850 nm. After the NFs dissolution in an aqueous media, c-BA-SLN maintained their physicochemical properties and zeta potential.

## 1. Introduction

Glioblastoma multiforme (GBM) is the most prevalent and aggressive brain tumor in adults, whose prognosis currently remains very poor (median survival less than 15 months). The intratumoral heterogeneity of GBM leads it to be classified by the World Health Organization (WHO) as a grade IV astrocytoma. The rapid proliferation and high capability of tumor cells to infiltrate healthy brain parenchyma explain the high rate of GBM recurrence and the lack of an effective therapeutic approach [1,2,3].

Nowadays, after surgical resection of the primary tumor, GBM patients are subjected to standard therapeutic regimens, including radiotherapy and adjuvant oral chemotherapy. Nevertheless, the GBM anatomical localization interferes with both its complete surgical excision and the chemotherapy success. In fact, the remaining tumor cells rapidly diffuse and form satellite tumors, particularly at the resection border margins, while the blood–brain barrier (BBB) prevents the achievement of therapeutic drug concentrations at the tumor site [4].

In the last decades, local intracranial delivery of chemotherapeutic agents has aroused considerable interest in the treatment of brain cancers; the implantation of drug-loaded bioresorbable systems at the tumor resection site allows the circumvention of the BBB to prevent systemic drug clearance and/or degradation, and to reduce systemic toxicity [5]. Polymeric nanofibers, endowed with a large surface-to-volume ratio and high porosity, have been proposed as valuable, implantable drug carriers for GBM therapy: they can provide a sustained drug release, ensuring high drug concentrations at the surgical resection area and, thus, minimizing post-surgery tumor recurrence [6]. In the literature, electrospinning has been recognized as a simple, low-cost, and versatile method of producing polymeric submicron fibers for the local and controlled release of anticancer drugs, such as paclitaxel [7,8], temozolomide [9,10,11], and carmustine [12,13], in brain cancer therapy.

Implantable electrospun nanofibers have also been proposed as valuable carriers for drug-loaded nanoparticles, whose internalization in the remaining glioma cells, after local administration in the resection cavity, could be limited by the high brain interstitial pressure and the possibility of being expelled from the target area [14].

The combination of nanofibers and nanoparticles to obtain composite nanosystems represents a novel therapeutic strategy that is already being investigated in the preparation of wound dressings [15] and the delivery systems for chemotherapeutic agents [16] and mRNA [17].

The use of implantable composite nanosystems in GBM therapy may permit to overcome the limitations involved in the local administration of drug-loaded nanoparticles, to improve their stability, and to further prolong and sustain drug release. In this scenario, Irani et al. have recently developed temozolomide-loaded nanoparticles, prepared with chitosan by ionic gelation, which were embedded into a poly(caprolactone diol)-based polyurethane (PCL-Diol-b-PU) nanofibrous implant [14].

Among the natural polymers used for the production of nano-sized drug delivery carriers, chitosan (CS) plays a pivotal role due to its biocompatibility, biodegradability, and low immunogenicity [18]. In cancer diagnosis and therapy, CS has been widely used not only as a nanoparticle-forming polymer, but also as coating material of polymeric, lipid, and magnetic nanosystems [19,20]. In particular, it has been recently demonstrated that nanoparticles consisting of, or decorated with, CS showed a selective cytotoxic effect against glioma cells [21,22]. Since the pH-dependent solubility of CS represents the main limit to the fabrication of drug-loaded systems, the reactive sites on glucosamine units of CS backbone (primary hydroxyl or amino, or both sites) could be chemically substituted with carboxymethyl groups. Carboxymethyl chitosan derivatives (CMCS), namely, O-, N-, and N,O-CMCS, show excellent solubility in an aqueous media, preserving similar properties with CS [18,23].

Given these premises, the aim of the present work was to develop a novel composite nanosystem (CN) as a potential tool for the local delivery of lipophilic anti-proliferative drugs in the treatment of GBM. Such a CN, composed of chitosan-coated solid lipid nanoparticles (c-SLN) embedded in O-carboxymethyl chitosan-containing nanofibers (NFs), was proposed to be implanted in the glioma resection cavity. c-SLN incorporation in electrospun NFs should permit a prolongation of their stability and easier handling and application in the tumor resection cavity. Moreover, after application, NFs should dissolve in the biologic fluids releasing the c-SLN, which should be taken up by the remaining tumor cells.

The work was articulated in three phases. In the first phase, SLN based on fatty acids (stearic acid, SA, or behenic acid, BA) were prepared by means of the coacervation method. This consists of the acidification of a hot micellar fatty acid sodium salt solution in the presence of poly(vinyl alcohol) (PVA) as a polymeric stabilizer. The influence of fatty acid type, pH, and PVA concentration on SLN physicochemical properties (particle size and polydispersity index) and zeta potential was investigated.

In the second phase, SLN characterized by the lowest particle size, functional to their incorporation in NFs, were coated with chitosan (c-SLN). For this purpose, two different coating approaches (one- and two-step) and three different polymer molecular weights (MWs) were considered. c-SLN were loaded with a fluorescent hydrophobic probe (Nile Red, NR) and their cytotoxicity and capability to be internalized in a human glioblastoma cell line (U-373) were investigated in comparison with CS-free SLN.

The third phase was focused on CN preparation and characterization. In particular, c-SLN were embedded in O-carboxymethyl chitosan-containing nanofibers produced by electrospinning. CN morphology and the capability to release c-SLN upon dissolution in an aqueous media was investigated.

## 2. Materials and Methods

### 2.1. Materials

For the preparation of lipid nanoparticles, the following materials were used: chitosan low molecular weight (LCS; MW = 50,000–190,000 Da; 20–300 cP, 1% *w*/*w* in 1% acetic acid, 25 °C, Brookfield; 75–85% deacetylated), chitosan medium molecular weight (MCS; MW = 190,000–310,000 Da; 200–800 cP, 1% *w*/*w* in 1% acetic acid, 25 °C, Brookfield; 75–85% deacetylated), chitosan high molecular weight (HCS; MW = 310,000–375,000 Da; 800–2000 cP, 1% *w*/*w* in 1% acetic acid, 25 °C, Brookfield; >75% deacetylated), poly(vinyl alcohol) (PVA; MW = 9000–10,000 Da; 80% hydrolyzed), and Nile Red (NR) were purchased from Sigma Aldrich (Milan, Italy), sodium stearate (Na-SA) was obtained from AAKON Polichimica Srl (Milan, Italy), sodium behenate (Na-BA) was obtained from Larodan, Research Grade Lipids (Monroe, MO, USA), and sodium hydroxide (NaOH 0.1M) and hydrochloric acid (HCl 1M) were obtained from Carlo Erba Reagents Srl (Milan, Italy).

All reagents used for cell cultures were purchased from Sigma Aldrich (Milan, Italy).

For the synthesis of O-carboxymethyl chitosan (O-CMCS), all the reactants and solvents were supplied by Sigma Aldrich (Milan, Italy).

For the preparation of electrospun nanofibers, the following materials were used: Kolliphor P407 poloxamer (P407, BASF SE, Ludwigshafen, Germany) and poly(ethylene oxide) high molecular weight (h-PEO, MW = 4000 kDa, Colorcon, Dartford, UK).

### 2.2. Preparation of Solid Lipid Nanoparticles

Stearic acid-based SLN (SA-SLN), containing 1% *w/w* sodium stearate (Na-SA) and different concentrations of PVA (Table 1), were prepared through the coacervation method, slightly modified as described by [24]. Briefly, PVA was added to a Na-SA aqueous dispersion and the mixture was heated under stirring (300 rpm) just above Na-SA Krafft point (≈75 °C) to obtain a clear solution. Subsequently, the coacervating solution (HCl 0.1 M) was added drop-wise: the coacervation was performed at different pH (from 5.5 to 1.5) in order to identify the optimum pH value for the production of SA-SLN with the desired characteristics. The resulting suspension was cooled to 15 °C under stirring to allow SLN precipitation.

Behenic acid-based SLN (BA-SLN), containing different sodium behenate (Na-BA) and PVA concentrations (Table 2), were prepared according to the above-mentioned method.

### 2.3. Preparation of Chitosan-Coated SLN (c-SLN)

Two different methods were considered for the preparation of SLN coated with chitosan (CS) (c-SLN).

In the first method, named one-step, c-SLN were prepared by coacervation (as described in Section 2.2) using HCl 0.1 M containing 0.25% *w*/*w* CS at low molecular weight (LCS) as the coacervating solution.

In the second method, named two-step, SLN dispersion, prepared as described in Section 2.2, was centrifuged at 12,000 rpm for 30 min (Ultracentrifuge type 60 Ti fixed-angle rotor; Beckam Coulter, Milan, Italy). After supernatant removal, SLN were dispersed under stirring for 24 h in CS 0.1% *v*/*v* acetic acid solution; SLN:CS *w*/*w* ratio was maintained equal to 50:1 [25]. In the two-step method, CS at three different molecular weights, low (LCS), medium (MMW), and high (HMW), were considered.

After centrifugation, SLN were dispersed in deionized MilliQ water and their electrical properties were investigated in order to confirm the successful PVA removal.

### 2.4. Characterization of SLN and c-SLN

#### 2.4.1. Particle Size, Size Distribution, and Zeta Potential

SLN and c-SLN particle size and polydispersity index (PDI) were measured by dynamic light scattering (DLS) (Litesizer 500, Anton Paar, Turin, Italy); each dispersion of SLN was diluted 1:9 *v*/*v* in filtered deionized MilliQ water and analyzed at a scattering angle equal to 90°. PDI indicates the width of the size distribution ranging between 0 (monodisperse system) and 1. Three replicates were performed for each sample.

SLN and c-SLN zeta potential was investigated by electrophoretic light scattering (ELS), which measured the speed of the SLN/c-SLN in the presence of an electric field (Litesizer 500, Anton Paar, Turin, Italy); each dispersion of lipid nanoparticles was diluted 1:9 *v*/*v* in filtered deionized MilliQ water. Three replicates were performed for each sample.

#### 2.4.2. Storage Stability Studies

A preliminary stability test was performed by analyzing particle size, PDI, and zeta potential of SLN after 7 days storage at 4 °C (Litesizer 500, Anton Paar, Turin, Italy).

#### 2.4.3. Morphological Analysis

The morphological analysis of SLN was performed by means of a transmission electron microscope (TEM) Jeol JEM-1200n EXIII (Jeol USA Inc., Peabody, MA, USA), equipped with TEM CCD camera Mega View III. The images were captured after deposition of 10 μL of fresh SLN dispersion, properly diluted 1:2500 *v*/*v* in filtered deionized MilliQ water, on a grid (Nickel Square Mesh-Grid, 300 mesh, 3.05 mm diameter; Agar Scientific, Essex, UK) previously coated with Collodion solution (Sigma Aldrich, Milan, Italy).

#### 2.4.4. Production Yield

The production yield was determined by a gravimetric test, slightly modified as described by [26]. In detail, SLN dispersion was centrifuged at 25,000 rpm for 30 min (Ultracentrifuge type 60 Ti fixed-angle rotor; Beckam Coulter, Milan, Italy); after supernatant removal, SLN were freeze-dried (Heto Dryer, Analytica De Mori, Milan, Italy) for 24 h and subsequently weighed (SLN weight). The production yield (Y%) was calculated according to the following equation:

Y% = (SLN weight/total solid weight) × 100(1)

where total solid weight corresponded to the weight of all the reagents (fatty acid sodium salt and PVA) used for SLN production.

#### 2.4.5. Encapsulation Efficiency

Regarding SLN encapsulation efficiency, both SLN and c-SLN loaded with a fluorescent hydrophobic probe (Nile Red, NR) were prepared and characterized as described in Section 2.2, Section 2.3 and Section 2.4 with a few modifications. Briefly, NR was dissolved in a minimal volume of absolute anhydrous ethanol (Carlo Erba Reagents Srl, Milan, Italy) and added to the hot fatty acid sodium salt aqueous dispersion in order to reach a final NR concentration equal to 6 μg/mL [27]. Subsequently, the coacervation was performed as previously described.

A known volume of NR-loaded SLN or c-SLN dispersion was centrifuged (25,000 rpm for 30 min); the supernatant containing non-encapsulated NR (free NR) was subjected to spectrofluorimetric analysis (LS50B, Perkin Elmer, Milan, Italy) at excitation and emission wavelengths of 554 and 638 nm, respectively. A standard curve was used to determine the NR concentration in the supernatant. The encapsulation efficiency (EE%) was calculated according to the following equation:

EE% = [(initial NR amount − free NR amount)/initial NR amount] × 100(2)

where the initial NR amount was the NR amount added to the starting micellar solution, while the free NR amount was the non-encapsulated NR amount quantified in the supernatant.

#### 2.4.6. Cytotoxicity Test

U-373 MG (Uppsala) cells, derived from a human glioblastoma astrocytoma, were purchased from the European Collection of Authenticated Cell Cultures (ECACC, Salisbury, UK). U-373 MG (from 7th to 15th passage) were cultured in Eagle’s Minimal Essential Medium (EMEM) supplemented with 10% heat-inactivated FBS, 2 mM glutamine, 1% non-essential amino acids, and 1 mM sodium pyruvate; cell cultures were maintained at 37 °C in 5% CO_2_-containing and 95% air atmosphere.

The cytotoxicity of SLN and c-SLN was assessed on U-373 cells; in particular, the mitochondrial dehydrogenase activity that reduces 3-(4,5-Dimethylthiazol-2-yl)-2,5-diphenyl-tetrazolium bromide (MTT, Sigma Aldrich, Merck KGaA, Darmstadt, Germany) was used to determine cell viability using a quantitative colorimetric assay [28].

U-373 cells were seeded overnight in 96-well plates at a density of 15 × 10^3^ viable cells/well, respectively. Cells were then exposed for 24 h to different SLN and c-SLN dispersions, diluted 1:100 v/v in complete culture medium.

After treatment, according to the experimental setting, cells were exposed to an MTT solution (1 mg/mL) in complete medium. After 4 h of incubation with MTT, cells were lysed with dimethyl sulfoxide (DMSO) and cell viability was quantified by reading absorbance at 570 nm wavelength, using Synergy HT multi-detection microplate reader (Bio-Tek, Winooski, VT, USA).

#### 2.4.7. Cell Uptake

U-373 cells were seeded onto poly-l-lysin-coated coverslips for 24 h before exposure to NR-loaded SLN and c-SLN for 6 h. Briefly, cells were fixed in ethanol 70% at −20 °C, washed with phosphate-buffered saline (PBS). Then, cells were rinsed in PBS and incubated for 10 min with Hoechst solution to counterstain the nuclei. After rinsing with PBS, cells were mounted upside-down on a glass slide in a drop of Mowiol mounting medium (Merck KGaA, Darmstadt, Germany). Cells were photographed with AxioCam MRc5 mounted on Zeiss Axioskop 40 microscopy (Oberkochen, Germany).

### 2.5. Preparation of Electrospun Nanofibers (NFs)

#### 2.5.1. Preparation of Polymeric Solutions for Electrospinning

Five polymeric solutions (OC1–OC5), containing 1.5% *w*/*w* h-PEO and increasing O-carboxymethyl chitosan (O-CMCS; see Supplementary Materials: Synthesis and Characterization of O-Carboxymethyl Chitosan) [29] concentrations were prepared in distilled water (Table 3); P407 was added at the concentration of 2%. Before electrospinning, the polymeric solutions were maintained under stirring overnight at room temperature.

#### 2.5.2. Electrospinning and Nanofiber Characterization

All the polymeric solutions, after characterization in terms of rheological and electrical properties (see Supplementary Materials: Characterization of the Polymeric Solutions for Electrospinning), were prepared using an electrospinning apparatus (STIKIT-40; Linari Engineering, Grosseto, Italy), equipped with a high-voltage power supply, a syringe pump (Razel Scientific, Saint Albans, VT, USA), and a collector plate, covered by aluminum foil.

The electrospinning process was performed at a fixed spinneret-collector distance (22 cm), applying a voltage equal to 25 kV, and maintaining controlled conditions of temperature and relative humidity (32–37 °C and 18–22%, respectively).

NF morphology was investigated by means of a scanning electron microscope (Tescan Mira3 XMU, Brno, Czech Republic) and NF size was measured using the imaging analysis program ImageJ 2.0 (net.imagej: imagej:2.0.0-rc-55, Java-based operating system, 2009, National Institutes of Health, Bethesda, MD, USA); thirty fibers were considered for each sample.

### 2.6. Preparation and Characterization of Composite Nanosystem (CN)

c-SLN dispersion (3 mL) was blended with the most promising polymeric solution containing O-CMCS, h-PEO, and P407 (7 mL) in order to obtain a c-SLN polymeric mixture in which the concentrations of the polymeric components (Table 3) were maintained unvaried. Such a mixture was characterized in terms of rheological and electrical properties (see Supplementary Materials) and then electrospun according to the process and the environmental conditions reported in Section 2.5.1.

NF morphology was investigated as described above (see Section 2.5.2).

A portion of CN (area 1 cm^2^) was placed in 2 mL of saline at 37 °C. After 5 min, the solution obtained was subjected to dynamic and electrophoretic light scattering.

### 2.7. Statystical Analysis

Whenever possible, experimental values of the various types of measurements were subjected to statistical analysis, carried out by means of the statistical package Statgraphics 5.0 (Statistical Graphics Corporation, Rockville, MD, USA). In particular, one-way ANOVA followed by post hoc Sheffé test and Dunnett’s multiple comparisons test were carried out.

## 3. Results and Discussion

### 3.1. Chitosan-Coated Solid Lipid Nanoparticles (c-SLN)

In the present work, coacervation was selected as a solvent-free method for the preparation of SLN containing fatty acids (stearic acid, SA, or behenic acid, BA) by acidifying a hot micellar solution of fatty acid sodium salts in the presence of an appropriate polymeric stabilizer. pH lowering was responsible for SLN precipitation due to proton exchange between the coacervating solution and fatty acid sodium salts [24,27,30,31]. Moreover, such a method should allow the entrapment of hydrophobic drugs within SLN by drug dissolution in the micellar solution prior to acidification.

PVA was selected as a steric stabilizer, functional in preventing SLN aggregation; its non-ionic nature renders it less sensitive to pH shifts. In the literature, it is reported that PVA could differently interact with fatty acid-SLN according to its hydrolysis degree (HD) and its molecular weight (MW); in particular, Battaglia et al. [27] demonstrated that SA-SLN mean diameter increased on increasing PVA HD, while it did not significantly change on increasing PVA MW. Considering that the aim of the present work was the production of fatty acid-SLN with a mean particle size below 250 nm, 80% hydrolyzed PVA 9000 (MW = 9000–10,000 Da) was used for the preparation of both SA-SLN and BA-SLN [27].

The effect of pH on the physicochemical properties of SA-SLN and BA-SLN was investigated; in particular, a preliminary study was performed to identify the optimum pH value to be reached for the production of SA-SLN and BA-SLN with the desired characteristics.

**Figure 1:**
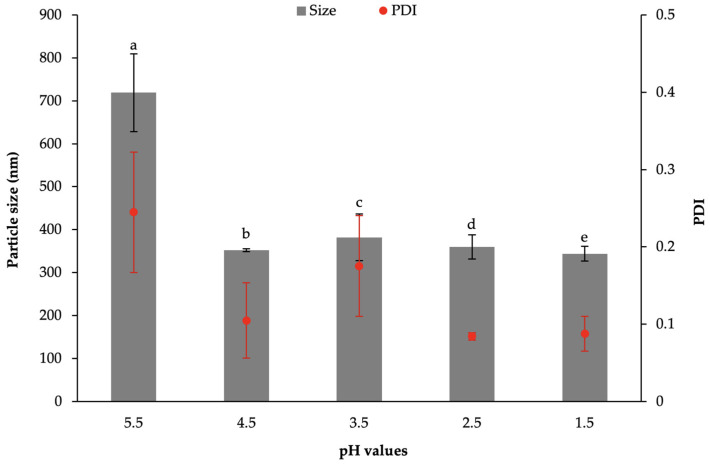
Particle size and PDI of S2-1 prepared by coacervation at different pH values. Data are expressed as mean ± SE (*n* = 6). ANOVA one-way; Sheffè Multiple Comparison (*p* < 0.05): a vs. b–e.

As shown in Figure 1, a pH lowering from 5.5 to 1.5 is accompanied by a reduction of both particle size and polidispersity index (PDI) of SA-SLN based on 1% *w*/*w* sodium stearate (Na-SA) and 2% PVA (S2-1); a similar trend was observed for SLN based on 1% *w*/*w* Na-SA and 3% PVA (S3-1) (data not shown).

**Figure 2:**
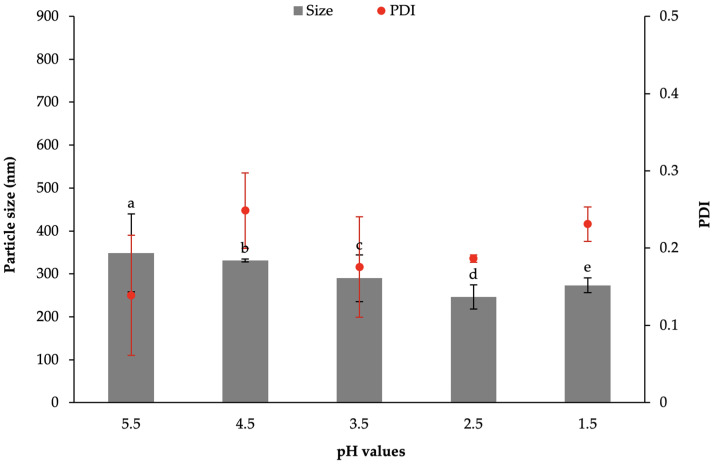
Particle size and PDI of B2-075 prepared by coacervation at different pH values. Data are expressed as mean ± SE (*n* = 6). ANOVA one-way; Sheffè Multiple Comparison (*p* < 0.05): a vs. c–e; b vs. c–e; c vs. d.

The physicochemical properties of BA-SLN, prepared in the presence of 0.75% *w*/*w* sodium behenate (Na-BA) and 2% *w*/*w* PVA (B2-075) and obtained at different pH values, are reported in Figure 2. A pH lowering below 4.5 produced a significant reduction in BA-SLN size; in particular, BA-SLN with dimensions lower than 250 nm and characterized by PDI < 0.2 are obtained at pH = 2.5.

According to the results obtained, both SA-SLN and BA-SLN were prepared by adding the coacervating solution (0.1 M HCl) drop-wise until pH ≅ 2.5 was reached before cooling. Table 4 shows the physicochemical properties and the zeta potential of SA-SLN and BA-SLN, respectively.

As regards to SA-SLN, containing 1% *w*/*w* Na-SA and different PVA concentrations (2% *w*/*w*, S2-1, and 3% *w*/*w*, S3-1), it can be noticed that the PVA concentration does not significantly influence SA-SLN mean sizes; moreover, both S2-1 and S3-1 show a negative surface charge due to the presence of SA carboxylic groups (Table 4).

As shown in Table 4, all the BA-SLN dispersions, containing 0.75% *w*/*w* Na-BA and different PVA concentrations (1% *w*/*w*, B1-075, 2% *w*/*w*, B2-075, and 3% *w*/*w*, B3-075), are monodisperse (PDI < 0.2) with a particle size below 350 nm and a zeta potential value ranging between −6 and −4 mV. Contrary to SA-SLN, it is noteworthy that an increase in the PVA concentration produces a significant decrease of the mean size of BA-SLN. Such a result could be explained by the fact that increasing PVA amounts may be necessary to stabilize SLN containing a long chain fatty acid, such as BA [27].

A preliminary stability test, performed after 7 days’ storage at 4 °C, shows no significant differences in SLN physicochemical properties and zeta potential (Table 4).

Among the colloidal dispersions prepared, BA-SLN containing 0.75% *w*/*w* Na-BA and 3% *w*/*w* PVA (B3-075) are characterized by the smallest size and, thus, selected for the continuation of the work. Sizes lower than 250 nm are desirable since SLN must be coated with a polymeric layer functional to their internalization in glioblastoma cells, and then loaded into polymeric nanofibers with a diameter ranging from 600 nm to 1000 nm.

**Figure 3:**
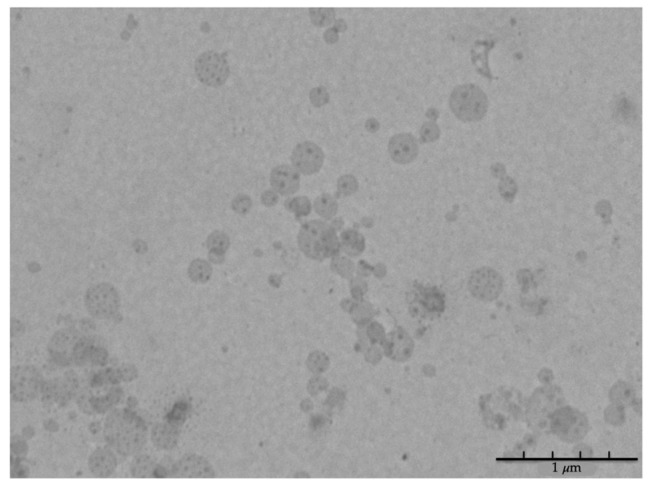
Transmission electron photo-micrographs of BA-SLN (scale bar: 1 μm).

TEM analysis was performed to investigate BA-SLN morphology: Figure 3 shows SLN characterized by a spherical shape and size lower than 250 nm, in line with the results obtained by DLS analysis.

Subsequently, BA-SLN were coated with CS, a natural polysaccharide widely used in the manufacturing of nano-scale drug delivery systems intended for the treatment of brain cancers [32]. The acid pH of the tumor environment ensures that CS chains are positively charged, thus able to interact with negatively charged phospholipid head groups or protein domains located in cell membranes. Therefore, the presence of CS as cationic coating polymer of SLN should promote an electrostatic interaction between coated SLN and the cell surface and their subsequent internalization [20]. Moreover, some authors have explained the role of CS in tumor targeting by its structural resemblance to hyaluronic acid that is a natural ligand for the CD44 receptor extensively expressed on cancer stem cell membranes [33].

In the present study, two different coating methods were proposed for the preparation of CS-coated BA-SLN (c-BA-SLN).

The first method involved a one-step process: 0.25% *w*/*w* CS at low MW (LCS) in HCl 0.1 M was used as coacervating solution. In Table 5, the physicochemical properties and the zeta potential of c-BA-SLN, prepared at pH ≅ 2.5, are compared to those measured for naked BA-SLN. This coating process produced an inversion of SLN surface charge; in particular, a significant increase in zeta potential value is recorded for c-BA-SLN, providing evidence of CS presence. Nevertheless, it can be observed that the one-step method produces a significant increase in particle size that is more easily attributable to SLN aggregation phenomena, rather than to CS deposition on a particle surface.

Even if the one-step coating represents a time-effective and simple method [20], c-BA-SLN failed to meet our expectations in terms of particle size; in fact, their size range appeared to be not quite suitable for loading into polymeric nanofibers.

Therefore, a second method involving a two-step process was studied: BA-SLN were coated with CS at three different MW (low, LCS, medium, MCS and high, HCS) by physical adsorption, more precisely by electrostatic interaction between the positively charged CS chains and the negatively charged BA-SLN surface due to the presence of BA carboxylic groups. In this second coating method, the centrifugation step, before the addition of CS, was crucial to PVA elimination and, thus, to the exposure of a greater number of negative charges on the BA-SLN surface capable of interacting with CS chains. The zeta potential of centrifuged BA-SLN, after supernatant removal and dispersion in deionized MilliQ water, was equal to −20.35 ± 0.51 mV, that is lower than the value of BA-SLN before centrifugation. This result highlights the successful PVA removal.

**Figure 4:**
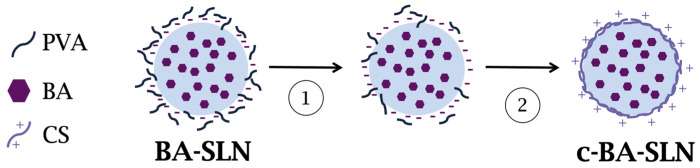
Schematic representation of the two-step coating method: (1) centrifugation at 12,000 rpm for 30 min; and (2) BA-SLN dispersion in CS solution for 24 h under mild magnetic stirring. Abbreviations: PVA: poly(vinyl alcohol); BA: behenic acid; CS: chitosan.

After centrifugation and PVA removal, BA-SLN were dispersed under stirring for 24 h in CS 0.1% *v*/*v* acetic acid solution, maintaining BA-SLN:CS ratio equal to 50:1 *w*/*w*, proposed in the literature as optimal (Figure 4) [25].

c-BA-SLN prepared by the two-step method are monodisperse and characterized by a particle size lower than 500 nm; both particle size and zeta potential provide evidence of CS coating (Table 5). As expected, CS MW significantly influenced the c-BA-SLN size and zeta potential; in particular, c-BA-SLN characterized by the lowest size are obtained in the presence of LCS (Table 5).

No significant differences between the physicochemical and electrical properties of BA-SLN coated with MCS and HCS are observed.

BA-SLN coated with LCS were selected for the prosecution of the work due to their particle size being lower than 350 nm. The production yield, calculated on the freeze-dried formulation, was around 60%.

Nile Red (NR) was used as a hydrophobic model substance; c-BA-SLN loaded with NR were characterized by a good EE% equal to 76%. The incorporation of NR did not produce significant changes in the BA-SLN physicochemical properties and zeta potential (data not shown).

**Figure 5:**
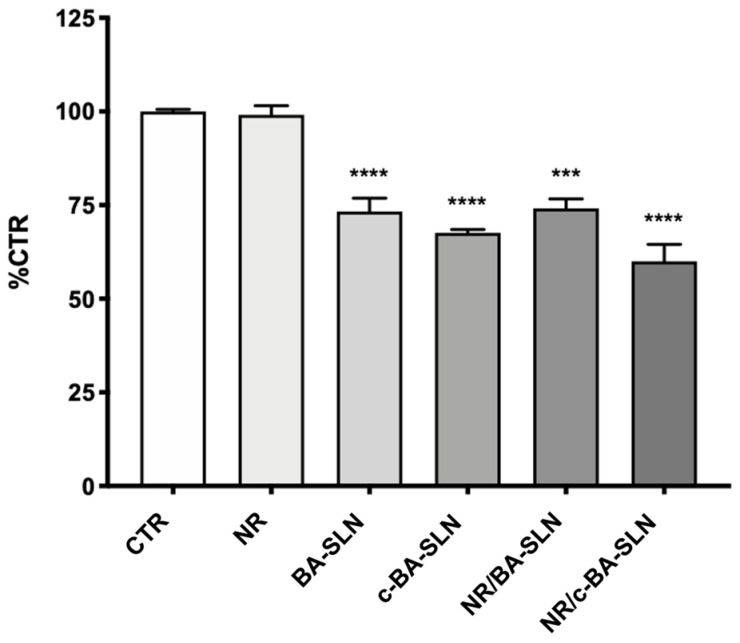
Study of the cytotoxic effect of BA-SLN and c-BA-SLN in human glioblastoma cells U-373. Cell viability was assessed by MTT assay after 24 h of treatments with BA-SLN and c-BA-SLN, both loaded and not with NR, at a dilution of 1:100. Results are expressed as %CTR ± SD. Dunnett’s multiple comparisons test; *** *p* < 0.001 and **** *p* < 0.0001 vs. CTR; *n* = 3.

The cytotoxicity of lipid nanoparticles, both CS-free (BA-SLN) and CS-coated (c-BA-SLN), was evaluated by MTT assay in human glioblastoma cell line U-373. In particular, U-373 cells were exposed to BA-SLN and c-BA-SLN, as well as to NR-loaded SLN (NR/BA-SLN) and NR-loaded c-SLN (NR/c-BA-SLN), for 24 h. As shown in Figure 5, the exposure of U-373 cells to BA-SLN and c-BA-SLN, both loaded and not with NR, induced a significant reduction in cell viability of about 30/40% with respect to the control (CTR), thus demonstrating a slight cytotoxic effect of SLN in this cell model. No effect on cell viability was observed in cells exposed only to NR.

Although it is recognized that small particles (<200 nm) were more rapidly taken up by cancer cells [34], it is also demonstrated that SLN composition and/or superficial decoration could strongly influence SLN internalization into cells, more than particle size [35].

**Figure 6:**
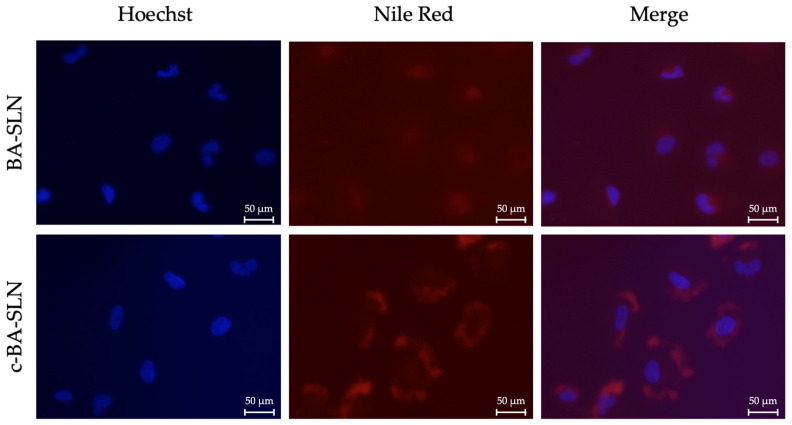
Internalization of NR/BA-SLN and NR/c-BA-SLN in human glioblastoma cells U-373. Human glioblastoma U-373 cells were exposed to NR/BA-SLN and NR/c-BA-SLN for 6 h. Immunocytochemistry was performed by using the red fluorescent dye Nile Red. Nuclei (blue) were stained by Hoechst. Magnification 40×. Scale bar: 50 μm.

The internalization of both NR/BA-SLN and NR/c-BA-SLN was assessed by fluorescence microscopy (Figure 6). In accordance with data from the literature reporting that nanoparticles composed of CS exhibit a high accumulation in cells compared to CS-free nanoparticles [21,22], a strong uptake of NR/c-BA-SLN was observed in U-373 cells after 6 h of treatments, as shown by immunocytochemistry experiments (Figure 6). In contrast, a lower internalization was observed for NR/BA-SLN, thus suggesting that CS coating significantly enhances BA-SLN uptake.

### 3.2. Electrsospun Nanofibers (NFs)

In the second phase of the work, electrospinning was selected as a simple, low-cost, and versatile technique [36,37] for the preparation of nanofibers (NFs) to be implanted in the glioma resection cavity as carriers for c-BA-SLN. In an attempt to produce polymeric NFs endowed with excellent solubility in an aqueous media, LCS was subjected to carboxymethylation of its hydroxyl moieties in order to obtain O-carboxylmethyl chitosan (O-CMCS) (see Supplementary Materials). Among carboxymethyl chitosan derivatives, O-CMCS was selected since it is water soluble in a wide pH range [38] and it has been recognized as an attractive polymer for the production of drug delivery systems intended for cancer therapy [18].

Increasing concentrations of O-CMCS were blended with fixed amounts of polyethylene oxide at high MW (h-PEO, 1.5% *w*/*w*) and poloxamer 407 (P407, 2% *w*/*w*). h-PEO and P407 were chosen on the basis of the results obtained in previous works [39,40]. PEO is a synthetic non-ionic polymer generally used as adjuvant to facilitate the electrospinning of chitosan and chitosan derivatives; it minimizes the repulsive charges between polycationic molecules and improves polysaccharide chain flexibility [41,42,43,44]. P407 was, instead, selected as surfactant able to promote polymeric solution ejection from the spinneret due to a reduction of solution surface tension [45].

**Figure 7:**
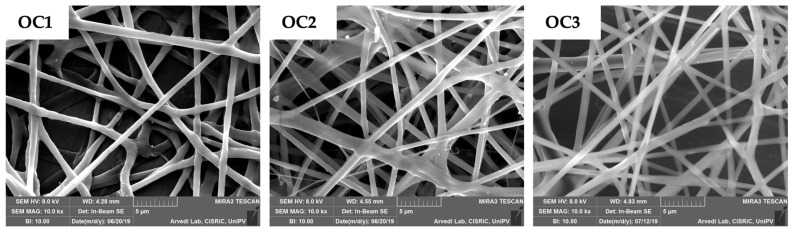
SEM micrographs of the electrospun O-carboxymethyl chitosan/h-PEO nanofibers obtained from the different polymeric solutions (22 cm distance; 25 kV voltage).

Polymeric solutions (OC1-OC5) were characterized in terms of rheological properties at increasing shear rates (see Supplementary Materials) and then electrospun. As an example, in Figure 7, SEM micrographs of OC1, OC2, and OC3 electrospun fibers (indicated with the same code used for the starting solutions) are reported. OC4 and OC5 solutions did not produce homogeneous fibers. OC3 fibers were chosen as a carrier for c-BA-SLN, since they are characterized by the highest size homogeneity (Figure 7; Table 6) and the best electrospinnability.

### 3.3. Composite Nanosystem (NC)

**Figure 8:**
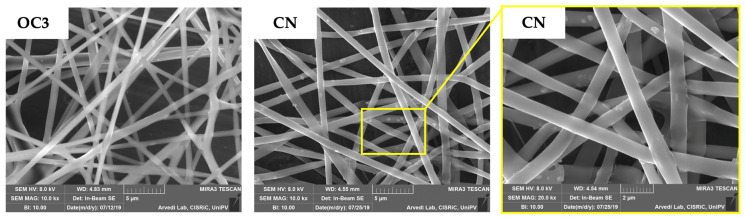
SEM micrographs of OC3 nanofibers and the composite nanosystem (CN), consisting of c-SLN embedded in OC3 nanofibers.

Figure 8 shows the composite nanosystem (CN) consisting of c-BA-SLN loaded in O-CMCS-containing nanofibers (OC3 NFs) produced by electrospinning; in the magnification on the right, it can be appreciated that c-BA-SLN are well-embedded in the matrix of polymeric fibers. After exposure to saline, NFs dissolved in a few minutes releasing the c-BA-SLN, which maintained their size and zeta potential (data not shown).

## 4. Conclusions

Homogeneous and negatively charged SLN, based on stearic or behenic acids (SA or BA, respectively), were prepared using a coacervation method at pH = 2.5, employing PVA as a stabilizer. BA-SLN containing 0.75% *w*/*w* BA sodium salt and 3% *w*/*w* PVA were selected for the prosecution of the work, being characterized with the smallest particle size and a PDI < 0.25; sizes lower than 250 nm are desirable since SLN must be coated with chitosan to promote their internalization in glioblastoma cells, and then loaded into polymeric nanofibers with a diameter ranging from 600 nm to 1000 nm.

BA-SLN were successfully coated with a chitosan (CS) at a low molecular weight by means of a two-step method that resulted in the removal of PVA through centrifugation and the subsequent CS physical adsorption on the SLN surface via electrostatic interaction between the positive chitosan chains and the negative SLN surface. In vitro testing performed on CS-free and CS-coated BA-SLN (c-BA-SLN), loaded with a fluorescent probe (Nile Red), evidenced a slight cytotoxic effect of SLN in U-373 human glioblastoma cells and the capability of c-BA-SLN to be internalized in this cell model to a greater extent compared to BA-SLN.

c-BA-SLN were profitably loaded in O-carboxymethyl chitosan-containing nanofibers by electrospinning to obtain a composite nanosystem able to dissolve upon application and to release the loaded c-SLN.

On the basis of the results so far obtained, we can conclude that the composite nanosystem that was developed is a promising candidate for the local delivery of lipophilic chemotherapeutic agents in the tumor resection site.

All the polymeric components of freely soluble NFs (O-CMCS, h-PEO, and P407) are biocompatible and biodegradable; regarding the fate of polymer degradation products, further studies will need to be performed.

A crucial issue in the case of implants/formulations that are locally administered in the brain is related to the fate of the biodegradation products, in particular, their**** capability to bypass the blood–brain barrier and reach the systemic circulation to be eliminated. Proper and deep in vitro and in vivo (animal model) investigations on the fate of such materials have to be carried out to verify the product safety and will be the object of further research.

## Figures and Tables

**Table 1:** SA-SLN composition, expressed as % *w*/*w*.

Formulation	Na-SA	PVA
S2-1	1	2
S3-1	1	3

**Table 2:** BA-SLN composition, expressed as % *w*/*w*.

Formulation	Na-BA	PVA
B1-075	0.75	1
B2-075	0.75	2
B3-075	0.75	3

**Table 3:** Composition of polymeric solutions for electrospinning, expressed as % *w*/*w*.

Solution	O-CMCS	h-PEO	P407
OC1	0.25	1.5	2
OC2	0.50	1.5	2
OC3	0.75	1.5	2
OC4	1.0	1.5	2
OC5	1.5	1.5	2

**Table 4:** Physicochemical properties (particle size and PDI) and zeta potential of SA-SLN and BA-SLN. Data are expressed as mean ± SE (*n* = 6). ANOVA one-way; Sheffè Multiple Comparison (*p* < 0.05): f vs. g; k vs. l; l vs. l’; c vs. d, e; d vs. e; e vs. e’; m vs. n; n vs. o, n’; o vs. o’.

Formulation	Time 0			7 Days after Preparation		
	Particle Size (nm)	PDI	Zeta Potential (mV)	Particle Size (nm)	PDI	Zeta Potential (mV)
S2-1	309.03 ± 13.85 ^a^	0.039 ± 0.009 ^f^	−2.17 ± 0.21 ^k^	326.55 ± 17.16 ^a’^	0.100 ± 0.029 ^f’^	−5.68 ± 0.25 ^k’^
S3-1	363.78 ± 31.73 ^b^	0.109 ± 0.025 ^g^	−4.44 ± 0.27 ^l^	338.25 ± 19.18 ^b’^	0.079 ± 0.029 ^g’^	−8.09 ± 0.27 ^l’^
B1-075	336.58 ± 5.69 ^c^	0.149 ± 0.022 ^h^	−6.05 ± 0.34 ^m^	323.62 ± 5.85 ^c’^	0.117 ± 0.027 ^h’^	−6.79 ± 0.38 ^m’^
B2-075	272.20 ± 10.44 ^d^	0.197 ± 0.044 ^i^	−4.00 ± 0.59 ^n^	271.87 ± 2.09 ^d’^	0.029 ± 0.019 ^i’^	−6.61 ± 0.61 ^n’^
B3-075	241.82 ± 5.94 ^e^	0.208 ± 0.055 ^j^	−6.15 ± 0.56 ^o^	268.20 ± 5.79 ^e’^	0.065 ± 0.028 ^j’^	−2.85 ± 0.22 ^o’^

**Table 5:** Physicochemical properties (particle size and PDI) and zeta potential of CS-free BA-SLN (BA-SLN), coated with CS at different MW (c-BA-SLN) according to two different methods. Data are expressed as mean ± SE (*n* = 6). ANOVA one-way; Sheffè Multiple Comparison (*p* < 0.05): a vs. b, d, e; b vs. c; c vs. d, e; a’’ vs. b’’–e’’; b’’ vs. c’’; c’’ vs. d’’, e’’.

Coating Method	Formulation	CS MW	Particle Size [nm]	PDI	Zeta Potential [mV]
	BA-SLN		241.82 ± 5.94 ^a^	0.208 ± 0.055	−6.15 ± 0.56 ^a’’^
One-step	c-BA-SLN	low	643.63 ± 134.25 ^b^	0.297 ± 0.116	31.79 ± 2.12 ^b’’^
Two-step	c-BA-SLN	low	330.40 ± 2.59 ^c^	0.144 ± 0.026	3.11 ± 0.31 ^c’’^
Two-step	c-BA-SLN	medium	457.00 ± 6.40 ^d^	0.277 ± 0.191	36.97 ± 3.19 ^d’’^
Two-step	c-BA-SLN	high	460.60 ± 5.39 ^e^	0.114 ± 0.048	45.64 ± 7.38 ^e’’^

**Table 6:** Mean diameter of electrospun nanofibers. Data are expressed as mean ± SE (*n* = 30). ANOVA one-way; Sheffè Multiple Comparison (*p* < 0.05): a vs. b,c.

Fibers	Diameter (μm)
OC1	0.57 ± 0.04 ^a^
OC2	0.70 ± 0.03 ^b^
OC3	0.85 ± 0.03 ^c^

## Data Availability

Data is contained within the Supplementary Materials.

## Supplementary Materials

The following are available online at https://www.mdpi.com/article/10.3390/polym13091371/s1: Figure S1: FTIR spectra of LCS (blue line) and its O-carboxymethyl derivative O-CMCS (green line), Figure S2: Viscosity values of OC1-OC5 polymeric solutions intended to be electrospun at 1000 s^−1^, and Figure S3: Flow profiles of OC3 and c-SLN/OC3 at increasing shear rates (1–1000 s^−1^).

## Abbreviations

BA	behenic acid
BA-SLN	behenic acid-based solid lipid nanoparticles
c-BA-SLN	chitosan-coated behenic acid-based solid lipid nanoparticles
CN	composite nanosystem
CS	chitosan
c-SLN	chitosan-coated solid lipid nanoparticles
DLS	dynamic light scattering
EE%	encapsulation efficiency
ELS	electrophoretic light scattering
GBM	glioblastoma multiforme
HCS	high molecular weight chitosan
HD	hydrolysis degree
h-PEO	high molecular weight poly(ethylene oxide)
LCS	low molecular weight chitosan
MCS	medium molecular weight chitosan
MW	molecular weight
Na-BA	sodium-behenate (or behenic acid sodium salt)
Na-SA	sodium-stearate (or stearic acid sodium salt)
NFs	nanofibers
NR	Nile Red
NR/BA-SLN	Nile Red-loaded behenic acid-based solid lipid nanoparticles
NR/c-BA-SLN	Nile Red-loaded chitosan-coated behenic acid-based solid lipid nanoparticles
O-CMCS	O-carboxymethyl chitosan
P407	Poloxamer P407
PDI	polydispersity index
PVA	poly(vinyl alcohol)
SA	stearic acid
SA-SLN	stearic acid-based solid lipid nanoparticles
SLN	solid lipid nanoparticles
TEM	transmission electron microscope
WHO	World Health Organization
Y%	production yield
