# Supplementary material for: A Composite Nanosystem as a Potential Tool for the Local Treatment of Glioblastoma: Chitosan-Coated Solid Lipid Nanoparticles Embedded in Electrospun Nanofibers
Source: Polymers (Basel). 2021 Apr 22;13(9):1371. doi: 10.3390/polym13091371 (PMC8122751; doi:10.3390/polym13091371)
Supplement: Supplementary file 1 [file polymers-13-01371-s001.zip › polymers-1186247-supplementary.pdf]

SUPPLEMENTARY MATERIALS

1. Synthesis and Characterization of O-Carboxymethyl Chitosan

1.1 Methods

O-carboxymethyl chitosan (O-CMCS) was prepared modifying the method described by [18,29]. The carboxymethylation was performed *via* stirring CS at low molecular weight (LCS, 5 g) in 100 ml of 20% NaOH (w/v) for 15 min. After that, monochloroacetic acid (15 g) was gradually added in the suspension and stirring was continued for 2.5 h at 40°C. The reaction was neutralized with HCl 37% in an ice bath and, subsequently, a solution of 70% methanol was added. The reaction mixture was washed with methanol and dried under reduced pressure for 5 h. Solvent evaporation was performed under reduced pressure by a Heidolph Laborota 4000 instrument (Heidolph Instruments GmbH & Co., Schwabach, Germany). Then, the sample was grounded with a blade mill (A10 IKA-Werke GmbH & Co., Staufen, Germany). Jasco ATR-IR-4100 was used to register the IR spectra applying the compound as powder.

1.2 Results and Discussion

Figure S1 shows the overlapping of FT-IR spectra of CS at low molecular weight (LCS) and the derivate O-carboxymethyl chitosan (O-CMCS). LCS displays two broad bands at 3445 and 3360 cm^{-1}, which are typical of NH_2 and OH stretching, and at 2919 cm^{-1} corresponding to CH stretching of methyl group. Two bands at 1655 cm^{-1} and 1596 cm^{-1} can be attributed to CO of NH-CO stretching and to NH bending, while at 1085 and 1030 cm^{-1} COH stretching bands are shown. On the other part, OCHT spectrum shows two new strong bands at 1580 and 1412 cm^{-1} attributable to symmetric and asymmetric stretching vibration of COO- salt. Thus, the introduction of carboxymethyl group was confirmed, suggesting that the substitution occurred at C6 position of chitosan. Data displayed here are in agreement with literature [18].

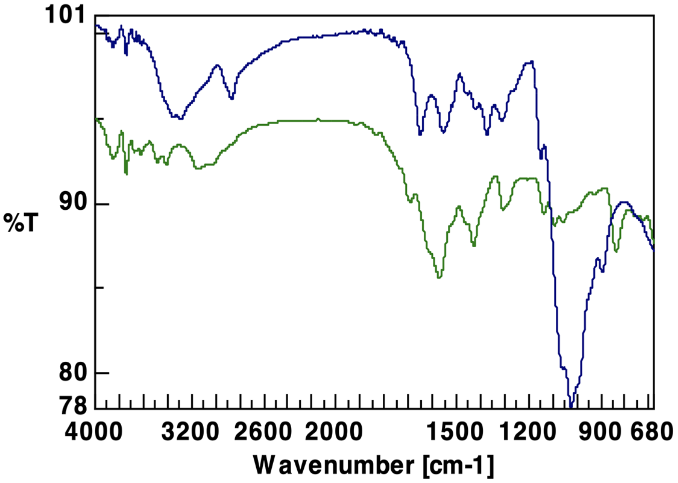

Figure S1. FTIR spectra of LCS (blu line) and its O-carboxymethyl derivative O-CMCS (green line).

2. Characterization of the Polymeric Solutions for Electrospinning

2.1 Methods

All the polymeric solutions (OC1-OC5) and the mixture of c-SLN dispersion and OC3 (c-SLN/OC3) were characterized in terms of rheological properties and conductivity, as reported in [39,40].

2.1.1 Rheological Measurements

Rheological analyses were carried out by means of a rotational rheometer (MCR 102, Anton Paar, Turin, Italy) equipped with a cone plate combination (CP50-1, diameter = 50 mm; angle = 1°) as measuring system.

Sample viscosity was investigated at increasing shear rates in the range 1–1000 s^{-1} at 33°; three replicates were effected for each polymeric solution. Three replicates were considered for each polymeric solution.

2.1.2 Conductivity Measurements

Sample conductivity was measured with Mettler ToledoTM FiveGoTM F3 conductivity meter apparatus (Fisher Scientific, Milan, Italy). Three replicates were considered for each solution.

2.2 Results and Discussion

In Figure S2, the results of the rheological analyses performed on OC1-OC5 polymeric solutions are reported. As expected, the viscosity value at 1000 s^{-1} increases on increasing O-CMCS concentration (Figure S2); such shear rate was selected since it simulated the stress to which the polymeric solutions were subjected during the electrospinning process.

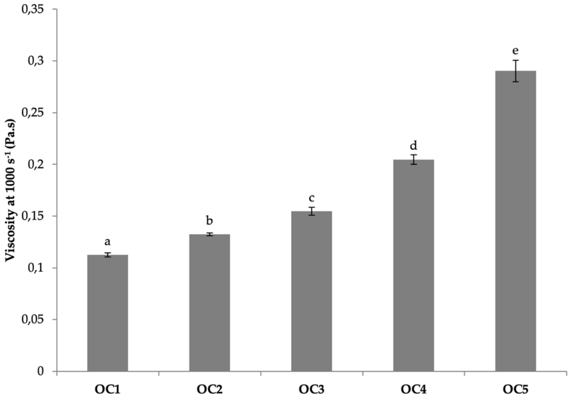

Figure S2. Viscosity values of OC1-OC5 polymeric solutions intended to be electrospun at 1000 s-1. Data are expressed as mean values ± SD (n=3). ANOVA one-way; Sheffè Multiple Comparison (p<0.05): a vs b-e; b vs c-e; c vs d-e; d vs e.

The blending of the c-SLN dispersion with OC3 (c-SLN/OC3), which was selected as the most promising polymeric solution for the production of homogenous nanofibers by electrospinning, did not alter the rheological properties of OC3; as shown in Figure S3, the flow profile of OC3 is comparable to that of c-SLN/OC3.

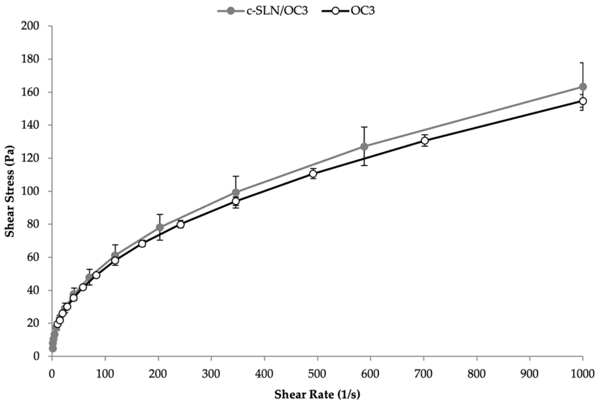

Figure S3. Flow profiles of OC3 and c-SLN/OC3 at increasing shear rates (1-1000 s-1). Data are expressed as mean values ± SD (n=3).

Noteworthy, c-SLN/OC3 was characterized by a conductivity value significantly higher than that of OC3 (2224.33 ± 54.24 µS/cm *vs* 1661.56 ± 37.89 µS/cm; mean value ± SD, n=3), probably due to the presence of c-SLN, whose surface was positively charged.
